# Student Classroom Misbehavior: An Exploratory Study Based on Teachers' Perceptions

**DOI:** 10.1100/2012/208907

**Published:** 2012-08-01

**Authors:** Rachel C. F. Sun, Daniel T. L. Shek

**Affiliations:** ^1^The University of Hong Kong, Faculty of Education, Hong Kong; ^2^Department of Applied Social Sciences, The Hong Kong Polytechnic University, Hong Kong; ^3^Public Policy Research Institute, The Hong Kong Polytechnic University, Hong Kong; ^4^Kiang Wu Nursing College of Macau, Macau; ^5^Division of Adolescent Medicine, Department of Pediatrics, University of Kentucky College of Medicine, Lexington, KY 40506, USA

## Abstract

This study aimed to examine the conceptions of junior secondary school student misbehaviors in classroom, and to identify the most common, disruptive, and unacceptable student problem behaviors from teachers' perspective. Twelve individual interviews with teachers were conducted. A list of 17 student problem behaviors was generated. Results showed that the most common and disruptive problem behavior was talking out of turn, followed by nonattentiveness, daydreaming, and idleness. The most unacceptable problem behavior was disrespecting teachers in terms of disobedience and rudeness, followed by talking out of turn and verbal aggression. The findings revealed that teachers perceived student problem behaviors as those behaviors involving rule-breaking, violating the implicit norms or expectations, being inappropriate in the classroom settings and upsetting teaching and learning, which mainly required intervention from teachers.

## 1. Introduction

Student misbehaviors such as disruptive talking, chronic avoidance of work, clowning, interfering with teaching activities, harassing classmates, verbal insults, rudeness to teacher, defiance, and hostility [1], ranging from infrequent to frequent, mild to severe, is a thorny issue in everyday classroom. Teachers usually reported that these disturbing behaviors in the classroom are intolerable [2] and stress-provoking [3], and they had to spend a great deal of time and energy to manage the classroom [4, 5]. Obviously, student misbehaviors retard the smoothness and effectiveness of teaching and also impede the learning of the student and his/her classmates. Moreover, research findings have shown that school misbehavior not only escalated with time but also lowered academic achievement and increased delinquent behavior [6, 7]. To lessen these immediate and gradual adverse effects of student misbehaviors, it is of primary importance to identify what exactly are these behaviors inside classroom.

In the literature, different terms have been used to describe problematic behaviors of students. For instance, Stewart et al. [8] referred student misconduct to disciplinary violations in school, for instance, tardiness, vandalism, fighting, stealing, and drinking on campus. When there are explicit rules and regulations in school and classroom, violation of these is apparently a “misbehavior or misconduct or discipline problem.” Nevertheless, a particular behavior is viewed as problematic may not necessarily be rule breaking, but inappropriate or disturbing in the classroom setting. For instance, daydreaming in class, not completing homework, talking in class, lesson disruption, bullying, and rudeness to the teacher are named as “problem behaviors” [9], “behavior problems,” [10, 11] or “disruptive behaviors” [4, 12]. These behaviors referred to “an activity that causes distress for teachers, interrupts the learning process and that leads teachers to make continual comments to the student” [13, page 60], or “the myriad activities which disrupt and impede the teaching-learning process” [14, page 43]. Noting that school misconduct is one of the manifests of the problem behavior syndrome [15–17], the term “problem behavior” was used to refer to all externalizing behaviors that violate explicit rules or implicit norms, disturb the classroom order, and irritate the process of teaching and learning in this study.

Several scales have been developed to measure teachers' perceptions of classroom problem behaviors. For instance, in the United Kingdom, Wheldall and Merrett [10] used ten items, including eating, nonverbal noise, disobedience, talking out of turn, idleness/slowness, unpunctuality, hindering others, physical aggression, untidiness, and out of seat, to measure behavior problems among primary school students. Houghton et al. [11] also used these behaviors to measure secondary school students' behavior problems, with a replacement of eating with verbal abuse because they found that teachers did not perceive eating as a problem behavior among secondary school students whereas verbal abuse was a more relevant behavior problem.

However, the cultural relevance of these scales to describe and measure disruptive behavior among primary and secondary school students in Hong Kong Chinese classroom is a concern that should be addressed. For example, Ho and Leung [12] and Leung and Ho [4] modified Wheldall and Merrett's scale [10] by dropping disobedience, and adding six student behaviors commonly reported by local teachers in Chinese school settings. These included verbal abuse, forgetfulness, nonattentiveness, gambling, reading other materials, and doing other things. However, as these descriptors of students' disruptive behaviors were formed almost a decade ago, their validity and applicability to Chinese classrooms nowadays may be questioned. Some student behaviors that have not be mentioned in the previous studies, such as daydreaming, sleeping, looking out of window, playing with personal stuff in private, bullying, disrespecting, talking back, arguing, quarrelling or fighting with teachers, complaining, and lack of independent initiative were found by a recent study in exploring Chinese teachers' perceptions of students' classroom misbehavior [18]. On top of this, uncooperativeness, emotional disturbance, overactivity and withdrawal were also reported as student classroom behavior problems by Chinese elementary school teachers [5]. Although these two studies were recent, both were conducted in mainland China. It is thus argued that the scales developed in these studies as well as the findings may be limited to describing student problem behaviors in mainland China classroom, which is different from the pluralistic classroom in which Confucian and Western teaching and learning approaches are used in Hong Kong. As such, direct employment of an existing scale is hardly sufficient to tap all the classroom problem behaviors exhibited by students. It is, therefore, important to carry out a qualitative research study to unravel relevant and up-to-dated descriptions of the students' problem behaviors in Hong Kong classroom based on the views of teachers.

Apart from exploring different categories of student problem behaviors inside classroom, it is also valuable to identify the common ones and the disruptive ones from the teachers' perspectives. Existing research findings showed that, among various types of student problem behaviors, “talking out of turn,” “hindering others,” and “idleness” were commonly reported by secondary school teachers as the most frequent and troublesome misbehaviors in the United Kingdom [11] and Australia [19]. Similar to these findings in the West, “talking out of turn” was rated by both primary and secondary school teachers as the most frequent and troublesome misbehavior, followed by “nonattentiveness” and “forgetfulness”—two other typical students' disruptive behaviors in Hong Kong classroom [4, 12]. In mainland China, “nonattentiveness”, “talking out of turn,” and “overactive” were reported as the most frequent and troublesome classroom behavior problems by the elementary school teachers in three provinces [5]. On the other hand, “daydreaming,” “talking out of turn,” and “playing with personal stuff” were rated as the most frequent classroom misbehaviors by a group of elementary, middle and high school teachers in another two provinces, while “daydreaming,” “slowness” and “talking out of turn” were the most troublesome classroom misbehaviors [18]. Apparently, “talking out of turn” is usually ranked as highly popular and disturbing student misbehavior across time and cultures and in different grade levels of students. With a specific focus on studying the problem behaviors of junior secondary students in Hong Kong classroom, this study attempted to replicate the previous studies in examining the problem behaviors perceived by teachers as the most common and disruptive. In addition, this study further attempted to investigate the most unacceptable problem behaviors in the eyes of teachers and the underlying reasons behind.

The primary goal of this study was to examine classroom problem behaviors among junior secondary school students in Hong Kong based on the views of teachers. The aims of this study were to (i) generate a list of categories of students' problem behaviors perceived by teachers in Hong Kong junior secondary school classroom, (ii) identify problem behaviors that were perceived as the most common, the most disruptive to teaching and learning in classroom, and the most unacceptable problem behavior and the reasons. Noting that the most frequent misbehavior can be somehow objectively observed, a particular behavior is regarded as the most disruptive or unacceptable depending on the teachers' subjective judgment and values, professional training, and years of teaching experiences. Therefore, this study recruited teachers with different years of teaching experiences and training background, in order to get a comprehensive view of the issue. It is a descriptive and exploratory qualitative research study. Academically, the present findings would add to the local literature, as recent research studies on this topic are scanty in Hong Kong [8, 9]. Even though there were some studies, they were conducted a decade ago [4, 12] and limited to focusing on the mainland China educational settings [5, 18]. Practically, it was expected that the findings would have profound importance to counseling and guidance work in the school context.

## 2. Methods

### 2.1. Participants

Three schools, each admitting students having low, medium or high academic competencies, were invited to join this study. In each school, four teachers who had experiences of teaching junior secondary grades (Grade 7, 8, and/or 9) and/or were members of the school counseling team and/or discipline teams were invited to join an individual interview. In total, twelve teachers (5 males and 7 females) participated in this study. Four of them were members of the school counseling team and three were members of the discipline team. The average of their teaching experiences was 9.25 years (range = 1–22 years). Their participation was voluntary and written consent from the school principals and the interviewees were obtained prior to data collection. Issues of anonymity and confidentiality in handling the data were also clearly explained at the beginning of each interview.

### 2.2. Instrument

A self-constructed semistructured interview guide was used for each individual interview. In the interview guide, questions and prompts used to explore the interviewees' perceptions of students' problem behaviors and their management strategies in the classroom and school contexts. The interviewees were asked to define “problem behaviors” based on their own understanding and interpretation. They were invited to use real-life examples to further illustrate their views. The average time for an interview was 49 minutes (range = 33–78 minutes). Each interview was conducted by two trained interviewers in Cantonese (the mother tongue of both the interviewers and interviewees). The interviews were audio-taped with informants' prior consent and transcribed in verbatim after the interview.

 As many questions were covered in the interview guide, only data related to the following questions were analyzed in this paper.

1. In the classroom, what student problem behaviors are there? Please list out as many as possible and describe.
2. Among these problem behaviors, which are the most common?
3. Among these problem behaviors, which are the most disruptive to teaching and learning?
4. Among these problem behaviors, which are the most unacceptable? Please illustrate.

### 2.3. Data Analysis

Findings pertinent to teachers' perceptions of students' problem behavior inside classroom are reported in this paper. Data was analyzed by using general qualitative analyses techniques [20]. First level of coding was conducted by a colleague who has a Bachelor degree of Psychology and teaching experiences. Semantically similar words, phrases, and/or sentences that formed meaningful units in each conclusion at the raw response level were grouped whereas semantically different data were divided. Further checking and second levels of coding and categorization were conducted by the first author, in which similar codes were grouped to reflect higher-order categories of theme. The coding and categorization were finalized with consensus among the coders and further checked by a colleague with a Bachelor degree of Psychology and professional counseling training.

As the code and categorization were inductively derived from the data, both intra- and interrater reliability on the coding were calculated to ensure the credibility of the findings. In the reliability test, 20 raw responses were randomly selected for each rater to code without referring to the original codes. The intrarater reliability tests were conducted by the two coders independently; whereas the interrater reliability tests were conducted by two colleagues (one has a Master degree and several years of teaching experiences and one has a Bachelor degree) independently. The reliability of the categorization was on the high side, because the intrarater agreement percentages were both 100%; while the interrater agreement percentages were 80% and 95%.

## 3. Results

### 3.1. Categories of Classroom Problem Behaviors

Table 1 summarizes 88 responses regarding students' problem behaviors inside classroom reported by 12 informants. The responses were classified into 17 main categories, and 6 of them were further divided into subcategories. As shown in Table 1, the problem behaviors reported by the teachers were mostly “doing something in private,” “talking out of turn,” “verbal aggression,” “disrespecting teachers,” “nonattentiveness/daydreaming/idleness,” “sleeping,” “habitual failure in submitting assignments,” and “out of seat”.

Teachers reported that students would do something in private which was unrelated to the lesson, such as reading, drawing, and doing other homework. Some teachers pointed out that it was a rising phenomenon that students liked to use electronic devices, such as mobile phone for texting people inside or outside classroom, playing electronic games, surfing webpage, or listening to music. In response to this phenomenon, there were regulations in some schools prohibiting students to switch on their mobile phones inside school.

“Talking out of turn” was another problem behavior which was mainly referred to students chatting among themselves on irrelevant topics that disrupts the lessons, calling out, and making remarks on somebody or something without teachers' permission. It is distinguished from “verbal aggression” which was referred to more hostile verbal expression, such as teasing, attacking, quarrelling, and speaking foul language.

“Disrespecting teachers” appeared to be an attitude, but the teachers could concretely describe some behaviors under this category. For instance, a teacher mentioned that refusing to follow instructions was a disobedient and disrespectful behavior. Teacher B02 commented that

“…challenging your (teachers') authority, mainly like, if you ask them not to do something, they are rebellious and insist to behave the other way round. They won't listen to teacher's opinion. They will insist to do what they think…These behaviors are mainly perceived in lower competent classes at the moment.”

Another teacher illustrated that disrespecting teachers meant rudeness, talking back, and confronting teachers. As remarked by Teacher C04:

“sometimes they will even dispute against their teacher…A student gave an irrelevant answer to teacher's question, that is, the teacher asked a serious question but the student gave a casual answer. If the teacher commented on, the student would be enraged and hostile, and then disputed against the teacher. Scolding teacher was unusual, unless the student was agitated. At the school level, I think there were less than five cases of scolding teacher in an academic year. Quite rare. When arguing, students usually had poor attitudes, especially boys. Hence, teachers would scold at them, and the students would become hostile, temper-losing… more seriously, they would knock tables or throw books to express their anger. But this situation was very rare; say one to two cases a year.”

“Nonattentiveness/daydreaming/idleness,” “sleeping,” and “out of seat” (including changing seats deliberately, wandering around the classroom, catching, running away from the classroom without permission) were commonly reported as problem behaviors inside classroom. Some teachers also regarded failure to submit assignments on time in a habitual manner as one of the problem behaviors, as reflected in the following narrative:

“[failure in submitting homework on time] is one of the problems if you are talking about student's misbehavior at school…this is quite a big problem in fact…There are a large proportion of students who fail to submit their homework on time, especially among Form 1 (Grade 7) student…Only half class can submit the homework on time if you set the deadline once. You need to chase after them for the homework…I think Form 1 (Grade 7) students are more likely to fail to submit their homework. In Form 2 (Grade 8), some classes can do better” (Teacher C03).

Some teachers added that some of the aforementioned problem behaviors, such as “talking out of turn” and “disrespecting teachers,” were commonly found among a specific group of students who had special education needs. A teacher mentioned that

“once I taught a student with SEN (Special Educational Needs) who had attention deficit… He had problems in getting along with his classmates. When other classmates had wrong answers, he would immediately call out and point out their mistakes. This in fact slightly affected the class” (Teacher C01).

Another teacher reported that

“I know that there are one or two SEN student(s) in every grade in our school. These students are quite disruptive. For example, they often have emotional disturbance, run away from classroom and sometimes fight against with their teachers” (Teacher B01).

### 3.2. Problem Behaviors That Were Most Common and Disruptive to Teaching and Learning

Among various classroom problem behaviors reported, comparatively more teachers pointed out that “having disruptive conversation” was a form of “talking out of turn,” which was the most common and the most disruptive to teaching and learning (see Table 1). A teacher explained that

“chatting during lesson affects teaching and learning most… Whereas other behaviors such as daydreaming only affect self-learning, chatting will alter the whole class atmosphere as well as class progress. I have to stop the chatting, otherwise I cannot teach and the students who chat will miss the content of the lesson. If I do nothing, other students will imitate and join the conversation…As the classroom is small, others can still hear even you talk in a low voice. Moreover, students are very attentive to the surroundings. So such chatting can be disruptive even you chat in a very low voice” (Teacher C04).

“Nonattentiveness/daydreaming/idleness” was the next common and disruptive problem behavior. A teacher explained that

“daydreaming during lesson will affect learning. If they are not attentive to the teacher, they have already missed some knowledge” (Teacher B04).

### 3.3. The Most Unacceptable Problem Behaviors inside Classroom

As indicated in Table 1, “disrespecting teachers” were rated by five teachers as the most unacceptable problem behavior. As revealed in the interviews, such behavior indicated that students lacked proper attitudes and values in interpersonal relationships as well as in their morality. Teacher C04 remarked that

“disputing against teachers is disrespecting teachers…Other misbehaviors are just behaviors. The underlying reasons of these behaviors are simple. For instance, chatting in the middle of lesson could take place because they feel bored; or they just pop up some ideas to share with their neighbors. However, if they argue back or disrespect their teachers, it is something related to their attitudes and values. So I think this is the biggest problem…Normally, they behave offensively against individual teachers, a certain kind of teachers including those who are too gentle or those who are rigid but not convincing.”

Another teacher added that

“[in confrontation]…some students like to twist the fact and shout their fallacy out loud to amuse their classmates. This is something that I cannot accept…It is obvious that he does not hold a point but still insists he is correct. I think this kind of behavior is unacceptable” (Teacher C03).

“Talking out of turn” and “verbal aggression” were also mentioned by teachers as unacceptable, because these behaviors disrupted the classroom order, which required teachers to spend time in managing classroom discipline and thus would adversely affect teaching. Among these verbal aggressive behaviors, teachers revealed that they could not accept students speaking foul language and teasing others, particularly insult would hurt the bullied.

Furthermore, individual teachers mentioned that “non-attentiveness/daydreaming/idleness,” “out of seat,” “habitual failure in submitting assignments,” “clowning,” and “passive engagement in class” as unacceptable, mainly because these behaviors would affect student learning and classroom atmosphere. For instance, in a teacher's perception of “non-attentiveness,” he expressed that

“if all students are unwilling or not motivated to learn, it will be very disastrous” (Teacher A01).

Another teacher explained why “out of seat” was unacceptable:

“if they sit still on their chairs, it is settled and they are less likely to have distracting behaviors or more severe problem behaviors. If they are out of seat, they may act out. There is a greater chance that they will distract other students and so the whole class. Therefore, I think this behavior is relatively unacceptable” (Teacher C01).

Another teacher showed his view on “passive engagement in class” by stating that

“… the most unacceptable behavior? I think it is inactive during lesson. To me, it is misbehavior although it is not obvious. If there are a number of passive students in my class, it is hard for me to teach them. No matter how and what I teach, they just do not want to learn. Compared with these inactive students, those who make noise in class are better. At least there is interaction even we argue” (Teacher A02).

## 4. Discussion

Based on the perspective of teachers, this study attempted to generate a list of categories of students' problem behaviors in Hong Kong junior secondary school classroom, and to identify the most common, disruptive and unacceptable student problem behaviors. As shown in Table 1, a list of 17 student problem behaviors was reported by the teachers, including doing something in private, talking out of turn, verbal aggression, disrespecting teachers, nonattentiveness/daydreaming/idleness, sleeping, out of seat, habitual failure in submitting assignments, physical aggression, copying homework, nonverbal communication, clowning, playing, lateness to class, eating/drinking, have not yet prepared textbook well, and passive engagement in class. Among them, the most common and disruptive misbehavior was talking out of turn, particularly in the form of disruptive conversation. The next one was nonattentiveness/daydreaming/idleness. The most unacceptable problem behavior was disrespecting teachers in terms of disobedience and rudeness, followed by talking out of turn, and verbal aggression. Teachers would consider these behaviors as intolerable when they disrupt teaching, affect student learning adversely, or suggest the fact that students do not have proper values and attitudes. These findings indicate that teachers are concerned about classroom learning and student development, and they expect that there are respect, obedience, order, and discipline in the classroom.

 There were some unique findings of this study, although most of the categories of problem behaviors identified are similar to those reported in the previous studies. First, “doing something in private” was regarded as a student problem behavior in secondary school classroom in Chinese cultural contexts [12, 18], while it was not included in some studies conducted in the West [11, 19]. In this category, on top of dealing with personal stuff, doing other homework, reading, and drawing that are unrelated to the lesson, this study showed that using electronic devices (e.g., mobile phone) for texting, playing games, surfing webpage, and listening to music were regarded as problematic nowadays. With particular focus to Hong Kong, mobile phones are popular among adolescents. As these electronic devices are multifunctional and audio-visual stimulating, some students would be tempted to use them for communication and fulfilling personal satisfaction even during lesson. Actually, doing something in private is an off-task behavior in which students are doing something irrelevant to classroom learning. Others, like nonattentiveness, idleness, and daydreaming were grouped together as a category of problem behaviors in this study because they were mentioned as related to the fact that students were tired, lazy, or lacking learning motivation. Sleeping was a single category, because it was an obvious off-task behavior and would be disruptive if students imitate each others.

Similar to most of the existing studies [10–12], “talking out of turn” included calling out, making remarks, and having disruptive conversation. All these referred to verbal disturbance in the lesson without teacher's permission. This conception is much wider than the narrow definition in Ding et al.'s study [18] where “talking out of turn” was simply referred to calling out answers without raising hands and being called upon by teachers. As usual, “talking out of turn” was rated by teachers as the most common and disruptive to teaching and learning. It was due to the fact that the noises are disruptive and teachers need to spend time to manage, otherwise, such behaviors would escalate in term of frequency and intensity and would be contagious. Another reason is that when compared to “nonattentiveness/daydreaming/idleness,” irrelevant chatting is more than an off-task behavior that adversely affects students' own learning. It is also a distracting behavior hampering others' learning in the same classroom.

Following talking out of turn, “verbal aggression” appeared to be a distinct problem behavior which was disruptive as well as hostile, such as speaking foul language as well as making offensive or insulting remarks to tease and assault classmates that further led to quarrelling or mutual attacking [11, 12]. All these might escalate to “physical aggression”, such as striking and pushing each others and destroying things in the classroom. The lack of sympathy or hostility involved in these aggressive behaviors was mentioned as intolerable as the teachers recognized the hurt involved. It reflected that caring was valued in the eyes of the teachers when they judged a behavior was problematic or not.

It is not surprising that “disrespecting teachers” was highlighted in this study as a kind of unacceptable problem behavior, because respect and obedience are the deeply rooted values in Chinese education. “Disrespecting teachers” embraced disobedience, that is, refusing or failing to carry out instructions [10–12], and rudeness, that is, talking back and arguing with teachers [18]. Sometimes, these behaviors would also be perceived as offensive to authority. These findings further demonstrated that these values are still strongly held in teacher expectations, and thus behaviors that fail to comply were pinpointed as disrespectful and the students were judged as lacking proper values and attitudes. The findings suggest that problem behaviors include those breaking explicit rules as well as those infringing implicit norms or expectations.

Apart from respect and obedience, order and discipline are essential elements of the Chinese classroom. Therefore, “out of seat,” “playing,” “clowning,” “lateness to class,” “eating/drinking,” “copying homework,” and “habitual failure in submitting assignments” were some common student problem behaviors perceived as disruptive to classroom order. The interviews revealed that on one hand, the teachers would like to have more control on the classroom order and discipline for not only easy management but also facilitating student learning. On the other hand, they would like students to have more self-control or self-discipline which is an important ingredient in learning. Moreover, “have not yet prepared textbook well” and “passive engagement in class” were some unique problem behaviors reported by the teachers in this study. It also reflected that some teachers expected students to get ready for the lesson and take an active role to learn throughout the lesson. If students were passive and not engaged, similar to daydreaming and not paying attention, teachers tended to regard students as irresponsible for their learning and even lacking learning motivation. Again, perception or labeling of problem behaviors results from the mismatches between the student behaviors and the social expectations. In short, the present findings indicated that student problem behaviors are not necessarily rule-breaking, but violating the implicit norms (e.g., the cultural values of respect, obedience, order, and discipline) or expectations (e.g., students can control their behaviors and be responsible for their own and others' learning). These problem behaviors are inappropriate in the classroom settings, as well as upsetting the classroom teaching and learning, which mainly require intervention from teachers.

Although some unique findings were observed in this study, there were some limitations involved. First, as only twelve teachers from three secondary schools were involved, representativeness of the findings should be viewed with caution. Second, as only teachers were interviewed, the findings may reveal the assumptions and biases of the teachers due to their social role as “teacher.” Therefore, it would be more comprehensive if the views of the students can be also included. Apart from looking at the categorization and descriptions of student problem behaviors, it would be more insightful if the antecedents of these behaviors or effective classroom management strategies could be explored in future. In particular, it would be exciting to see how curricular-based programs can help to reduce classroom misbehavior. One example that should be considered is the Project P.A.T.H.S. (Positive Adolescent Training through Holistic Social Programmes) in Hong Kong [21]. There are findings showing that the program could promote holistic youth development and reduce adolescent substance abuse and delinquent behavior [22–24]. It would be interesting to see whether the program can lessen classroom misbehavior in the long run.

## Figures and Tables

**Table 1:** A Summary of the teachers' perceptions of student problem behaviors inside classroom.

Category	Subcategory	Number of responses	Number of responses regarding on the most common problem behavior	Number of responses regarding on the most disruptive behavior	Number of responses regarding on the most unacceptable problem behavior
	Dealing with personal stuff	3	0	0	0
	Doing homework	2	0	0	0
Doing something in private	Using electronic device (for texting, playing games, surfing webpage, listening to music)	4	0	0	0
	Irrelevant reading	2	0	0	0
	Irrelevant drawing	2	0	0	0

	Subtotal	13	0	0	0

Talking out of turn	Calling out	1	0	0	1
	Making remarks	1	0	0	0
	Having disruptive conversation	9	5	2	2

	Subtotal	11	5	2	3

Verbal aggression	Teasing classmates	4	0	0	1
	Attacking classmates	3	1	1	0
	Quarrelling with classmates	1	0	0	0
	Speaking foul language	2	0	0	1

	Subtotal	10	1	1	2

Disrespecting teachers	Disobedience/Refusing to carry out instructions	4	0	0	2
	Rudeness/Talking back, arguing with teacher	4	1	1	3

	Subtotal	8	1	1	5

Non-attentiveness/Daydreaming/Idleness		7	2	2	1
Sleeping		6	0	1	0

Out of seat	Changing seats	1	1	0	0
	Wandering around the classroom	2	0	1	1
	Catching	1	0	0	0
	Running away from the classroom	1	0	0	0

	Subtotal	5	1	1	1

Habitual failure in submitting assignments		5	0	0	1

Physical aggression	Striking classmates	2	0	0	0
	Pushing classmates	1	0	0	0
	Destroying things	1	0	0	0

	Subtotal	4	0	0	0

Copying homework		4	1	0	0
Non-verbal communication	Via body language, facial expressions, papers	4	0	0	0
Clowning		3	0	0	1
Playing		3	0	0	0
Lateness to class		2	0	0	0
Eating/Drinking		1	1	0	0
Have not yet prepared textbook well		1	0	0	0
Passive engagement in class		1	0	0	1

Total responses		88	12	8	15
